# Assembly of Colloidal Clusters Driven by the Polyhedral Shape of Metal–Organic Framework Particles

**DOI:** 10.1021/jacs.1c05363

**Published:** 2021-08-12

**Authors:** Yang Liu, Jiemin Wang, Inhar Imaz, Daniel Maspoch

**Affiliations:** † Catalan Institute of Nanoscience and Nanotechnology (ICN2), CSIC, and Barcelona Institute of Science and Technology, Campus UAB, Bellaterra, 08193 Barcelona, Spain; ‡ ICREA, Pg. Lluís Companys 23, Barcelona, 08010, Spain

## Abstract

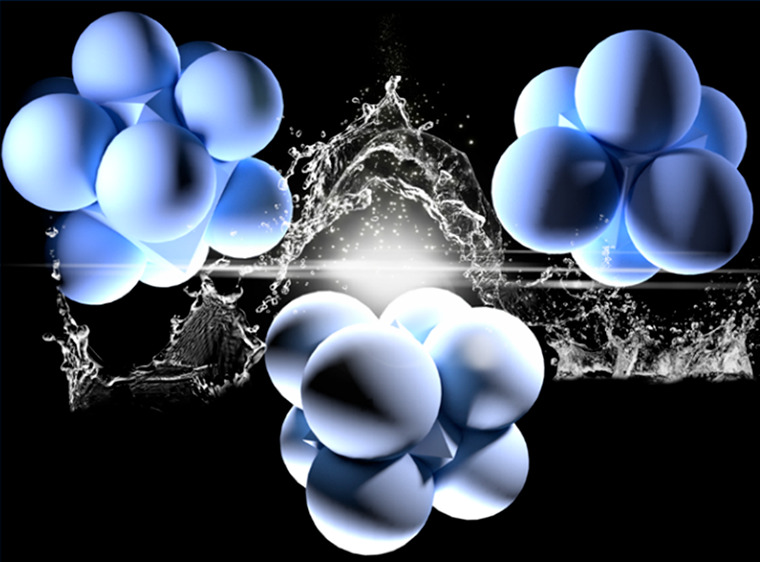

Control of the assembly of colloidal particles into discrete or higher-dimensional architectures is important for the design of myriad materials, including plasmonic sensing systems and photonic crystals. Here, we report a new approach that uses the polyhedral shape of metal–organic-framework (MOF) particles to direct the assembly of colloidal clusters. This approach is based on controlling the attachment of a single spherical polystyrene particle on each face of a polyhedral particle via colloidal fusion synthesis, so that the polyhedral shape defines the final coordination number, which is equal to the number of faces, and geometry of the assembled colloidal cluster. As a proof of concept, we assembled six-coordinated (6-c) octahedral and 8-c cubic clusters using cubic ZIF-8 and octahedral UiO-66 core particles. Moreover, we extended this approach to synthesize a highly coordinated 12-c cuboctahedral cluster from a rhombic dodecahedral ZIF-8 particle. We anticipate that the synthesized colloidal clusters could be further evolved into spherical core–shell MOF@polystyrene particles under conditions that promote a higher fusion degree, thus expanding the methods available for the synthesis of MOF–polymer composites.

The assembly of colloidal molecules or clusters^1−5^ is a crucially important step in the design of more sophisticated architectures such as patchy particles^6−8^ and self-assembled colloidal crystals^9^ (*e.g*., with diamond or pyrochlore structures) and opens novel avenues for the formation of new photonic crystals,^10^ micromotors,^11^ and drug-delivery systems.^12^ Recently, great progress has been made in the development of synthetic strategies to form such particle clusters.^13^ These strategies are mainly based on either the growth of particles on the surface of a preformed particle, via phase-separation phenomena or surface nucleation and growth, or the controlled assembly of presynthesized particles via attractive interactions such as DNA hybridization, electrostatic interactions, and/or van der Waals forces.^14^ These synthetic methods typically use spherical particles, such as silica, polymers, and inorganic particles. Consequently, they all must accomplish the difficult task of directing the assembly and/or growth of particles on the surface of isotropic spherical particles via methods such as tuning the size ratio between the particles or phase-separation phenomena to control the coordination number and geometry of the synthesized colloidal cluster. Building this type of clusters is therefore not an easy task, especially for those involving geometries with high coordination numbers, in which the assembly or growth of many particles must be controlled.

Herein, we propose a new strategy for controlling colloidal assembly, based on replacing the spherical particles with polyhedral ones, whose faces are used to direct both the position and number of assembled satellite polystyrene (PS) particles (Figure 1). The polyhedral particles thus act as core particles with predetermined “instructions” that direct the coordination number and geometry of the synthesized colloidal clusters. For example, an octahedral core particle would direct the formation of an eight-coordinated (8-c) cubic colloidal cluster, or a cubic core particle would template the synthesis of a 6-c octahedral colloidal cluster. In this way, polyhedral particles with a high number of faces could ideally be used to form clusters exhibiting geometries with high coordination numbers.

**Figure 1:**
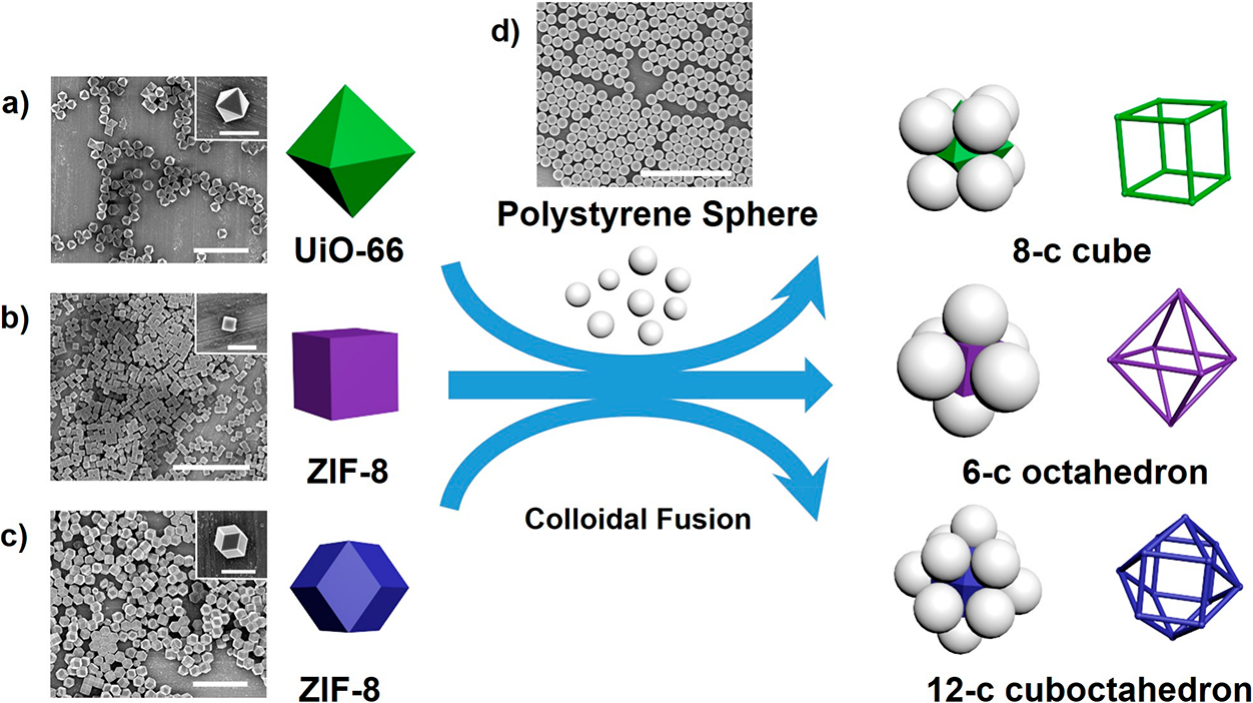
Schematic illustration of the use of polyhedral MOF crystals as core particles to assemble coordination clusters and the way in which their polyhedral shape dictates their coordination number and geometry. Using this strategy, colloidal 8-c cubic, 6-c octahedral, and 12-c cuboctahedral clusters were synthesized using octahedral UiO-66 particles, cubic ZIF-8 particles and rhombic dodecahedral ZIF-8 particles, respectively. Scale bars: 5 μm (parts a, b, c, d), 1 μm (insets in parts a, c), and 500 nm (inset in part b).

To implement our colloidal strategy, we selected metal–organic framework (MOF) particles as the core polyhedral particles. MOFs are a widely known class of porous crystalline materials that exhibit very high surface areas and have found a broad variety of applications, including gas storage and separation as well as catalysis and contaminant removal.^15,16^ However, in the present work, we targeted MOFs mainly because they are an excellent source of crystalline particles (Figure 1a–c) covering most known polyhedral shapes, thanks to the discovery of thousands of MOFs during the last 25 years.^17−19^ Moreover, recent advances in MOF synthesis allow them to be obtained as colloidal particles with highly homogeneous size and shape.^20−23^ Furthermore, their particle size can easily be tuned from ∼50 nm to ∼1 μm.^24−27^

Taking advantage of these properties, we report herein the use of polyhedral MOF particles to direct the assembly of spherical polystyrene particles in terms of both number and position, allowing the synthesis of colloidal clusters through mix-and-melt or colloidal fusion synthesis (Figure 1). This synthesis has previously been reported by Sacanna et al.^10,28,29^ and is based on the use of liquid oil droplets or inorganic nanoparticles as core particles onto which oppositely charged colloidal particles are stochastically aggregated. A plasticizer is then added to fuse the particles to form stable colloidal clusters, patchy particles, or core–shell particles.

To demonstrate the feasibility of our proposed strategy, we initially targeted the synthesis of 8-c cubic colloidal clusters using octahedral UiO-66 core particles (Figure 1a). The octahedral UiO-66 particles were first synthesized by heating a solution of ZrCl_4_, terephthalic acid, and acetic acid in DMF at 120 °C for 12 h. The synthesized particles were then collected by centrifugation, cleaned with DMF and methanol, and finally dispersed in water containing polyvinylpyrrolidone. Field-emission scanning electron microscopy (FESEM), X-ray powder diffraction (XRPD), and zeta-potential measurements of the resulting colloid revealed the formation of homogeneous octahedral UiO-66 particles with an edge size of 735 ± 21 nm (diameter: 1039 ± 30 nm) and a surface charge of approximately +45 mV (Figures S1,S2). Commercial aqueous colloids of spherical sulfonated polystyrene particles with diameters of 400 nm, 700 nm, and 1 μm as well as a surface charge of approximately −30 mV were used as satellite particles (Figure 1d).

In a typical experiment, colloidal clusters were assembled by adding 100 μL of the polystyrene colloid on top of 100 μL of the dispersion of UiO-66 particles. Then, tetrahydrofuran (THF), which acts as the plasticizer, was added to the mixture to give a final THF concentration of 18% (v/v), and the mixture was mixed by hand for 10 s. After this short period, the assembled colloidal clusters were isolated using density gradient centrifugation (10–30 wt % sucrose in water), washed with water, and finally dispersed in water.

Following this protocol, the assembly of spherical polystyrene particles with various diameters (400 nm, 700 nm, and 1 μm) was first systematically studied to determine the optimum particle size for the attachment of a single polystyrene particle on each triangular facet of the UiO-66 octahedron. For this set of experiments, the concentrations of polystyrene and the UiO-66 colloid were kept constant at 160 and 2 mg/mL, respectively (i.e., w_PS_:w_UiO-66_ = 80:1). Under these conditions, the 700 nm diameter polystyrene spheres proved to be ideal for individual particle assembly. The use of spherical polystyrene particles with a diameter of 400 nm resulted in the attachment of two or more particles on some facets of the octahedra, thus preventing the templating of cubic colloidal clusters by the polyhedral shape of the MOF particle (Figure S3). The 1 μm diameter polystyrene particles did not result in high occupancies of the eight facets of the octahedra, which was attributed to steric hindrance among themselves (Figure S4).

The synthesis of cubic colloidal clusters requires the attachment of a single polystyrene particle on each of the eight facets of the UiO-66 octahedra, i.e., achieving a coordination number of eight. Clusters missing one or more of the eight polystyrene particles can thus be considered defective. To maximize the formation of assemblies with eight polystyrene spheres on each UiO-66 particle, we performed a series of syntheses in which the w_PS_:w_UiO-66_ ratio was systematically varied (Figure 2, S5–S8). Figure 2e shows the statistical distribution of the coordination number of clusters synthesized using w_PS_:w_UiO-66_ ratios of 40:1, 80:1, 150:1, and 300:1. At a ratio of 40:1, the population of cubic colloidal clusters was 35%. However, in this case, most of the clusters were defective with coordination numbers of five, six, and seven, whereby the latter exhibited the highest population (44%). When the ratio was increased to 80:1, cubic clusters began to predominate (51%). At this ratio, the formation of defective clusters with coordination numbers of five and six was low, whereas those with a coordination number of seven represented 38% of the total population. This tendency continued at a ratio of 150:1, which provided the optimum conditions for the synthesis of cubic clusters. Under these conditions, 93% of the population consisted of perfect cubic colloidal clusters (61%; Figure 2a–c) or defective cubic clusters missing only one polystyrene sphere (32%; Figure 2d). Here, dynamic light scattering measurements further confirmed the formation of the clusters having a mean diameter of approximately 1.5 μm (Figure S9), which match those measured by FESEM (edge size: 1.34 ± 0.06 μm; diagonal size: 1.62 ± 0.08 μm). The use of higher ratios did not improve the formation of cubic colloidal clusters, but resulted in similar population percentages.

**Figure 2:**
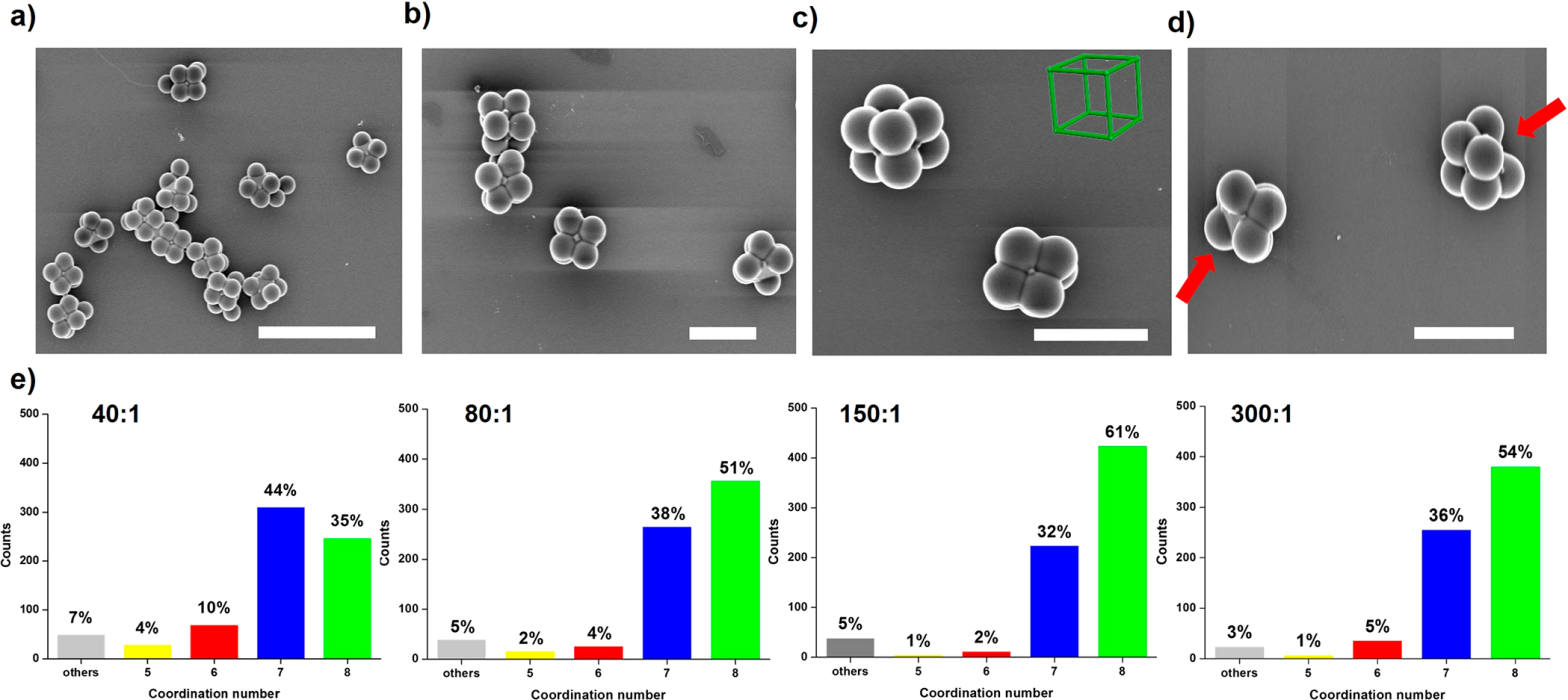
(a,b) FESEM images of the colloidal cubic clusters synthesized at w_PS_:w_UiO-66_ = 150:1. (c) FESEM image of two cubic clusters positioned in different orientations. (d) FESEM image of two defective colloidal clusters with a coordination number of 7; arrows highlight the missing polystyrene sphere. (e) Statistical distributions of the coordination number for clusters with different w_PS_:w_UiO-66_ ratios. Scale bars: 5 μm (part a) and 2 μm (parts b–d).

Having demonstrated the utility of the polyhedral shape of the core particles to serve as templates and guide the formation of cubic colloidal clusters, we extended the use of polyhedral MOF particles to form colloidal clusters with an octahedral geometry (coordination number: six) and with a much higher coordinated cuboctahedral geometry (coordination number: 12). To this end, we selected ZIF-8 particles due to their ability to be isolated as both cubic and rhombic dodecahedral colloidal particles (Figure 1b,c). According to our strategy, the attachment of a single polystyrene sphere to the six facets of ZIF-8 cubes and the 12 facets of rhombic dodecahedral ZIF-8 particles should lead to the formation of colloidal octahedra and cuboctahedra, respectively. Initially, ZIF-8 cubes were formed by incubating an aqueous solution of Zn(NO_3_)_2_·6H_2_O, 2-methylimidazole, and hexadecyltrimethylammonium bromide (CTAB) at room temperature for 5 h. Rhombic dodecahedral ZIF-8 particles were synthesized by incubating an aqueous solution of zinc acetate and 2-methylimidazole for 24 h. In both reactions, the particles were collected by centrifugation, washed with water, and redispersed in the presence of CTAB. FESEM and XRPD measurements confirmed the formation of cubic and rhombic dodecahedral ZIF-8 particles with edge dimensions of 205 ± 10 nm and 526 ± 27 nm, respectively (Figures S10–S13). Zeta-potential measurements of either particles showed a surface charge of approximately +40 mV. For the assemblies, we used negatively charged polystyrene particles with diameters of 200 and 600 nm in combination with the cubic and rhombic dodecahedral ZIF-8 particles, respectively, to optimize the attachment of a single polystyrene particle on each face of the ZIF-8 particles. Both 6-c octahedral (Figure 3a–c) and 12-c cuboctahedral (Figure 3d–f) colloidal clusters were then assembled following a similar process to that used for the assembly of the 8-c cubic clusters. It should be noted here that the purification step in these assemblies involved multiple sedimentation–redispersion processes, as density gradient centrifugation (10–30 wt % sucrose in water) caused etching of the ZIF-8 particles (Figure S14).

**Figure 3:**
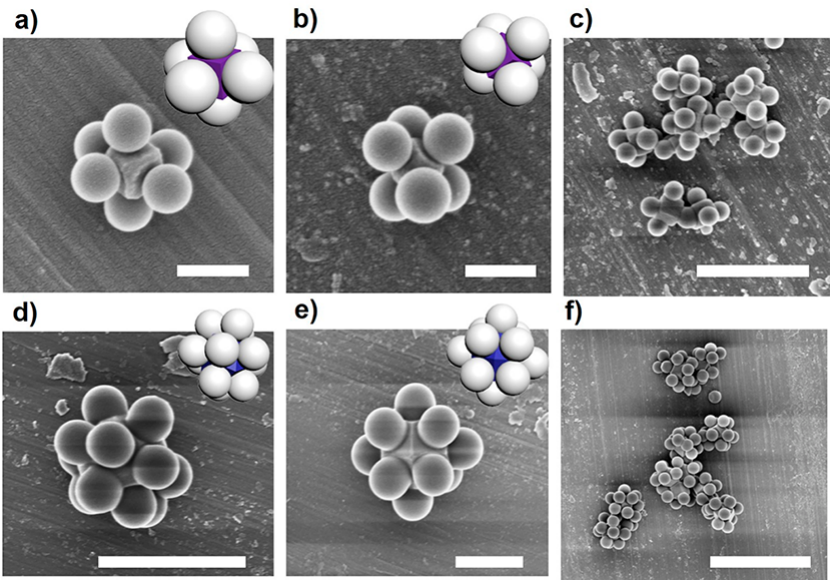
Schematic illustrations and corresponding FESEM images of 6-c octahedral (a–c) and 12-c cuboctahedral (d–f) clusters. Scale bars: 5 μm (part f), 1 μm (parts c–e), and 300 nm (parts a,b).

Synthesis by colloidal fusion is a method that provides access not only to colloidal clusters but also to patchy and core–shell particles. The latter type of particles can be synthesized by controlling the amount of plasticizer (in this case: THF) added to the reaction. Indeed, the melting process in these reactions is usually controlled by tuning the concentration of the plasticizer. As described above, stable colloidal molecules can be formed when low concentrations of the plasticizer are used. However, the addition of higher concentrations of plasticizer allows further melting of the particles to form core–shell or patchy particles.^29^ In conjunction with the use of MOFs as core particles, this possibility opens the door for the creation of MOF@polymer composites in which one MOF particle is encapsulated in a polymer shell (Figure 4). Accordingly, the above-described synthesis of 8-c cubic clusters was repeated with the amount of THF increased to 26% or 30% (v/v). FESEM images of the resulting assemblies showed that the cubic clusters evolved into cellular-type particles when the THF concentration was increased to 26% v/v (Figure 4a,b). In these particles, the eight polystyrene spheres merged with one another and engulfed the core UiO-66 particle. This melting process was completed at 30% v/v THF, where spherical core–shell particles consisting of a single UiO-66 particle encapsulated in a polystyrene shell were formed (Figure 4a,c–e).

**Figure 4:**
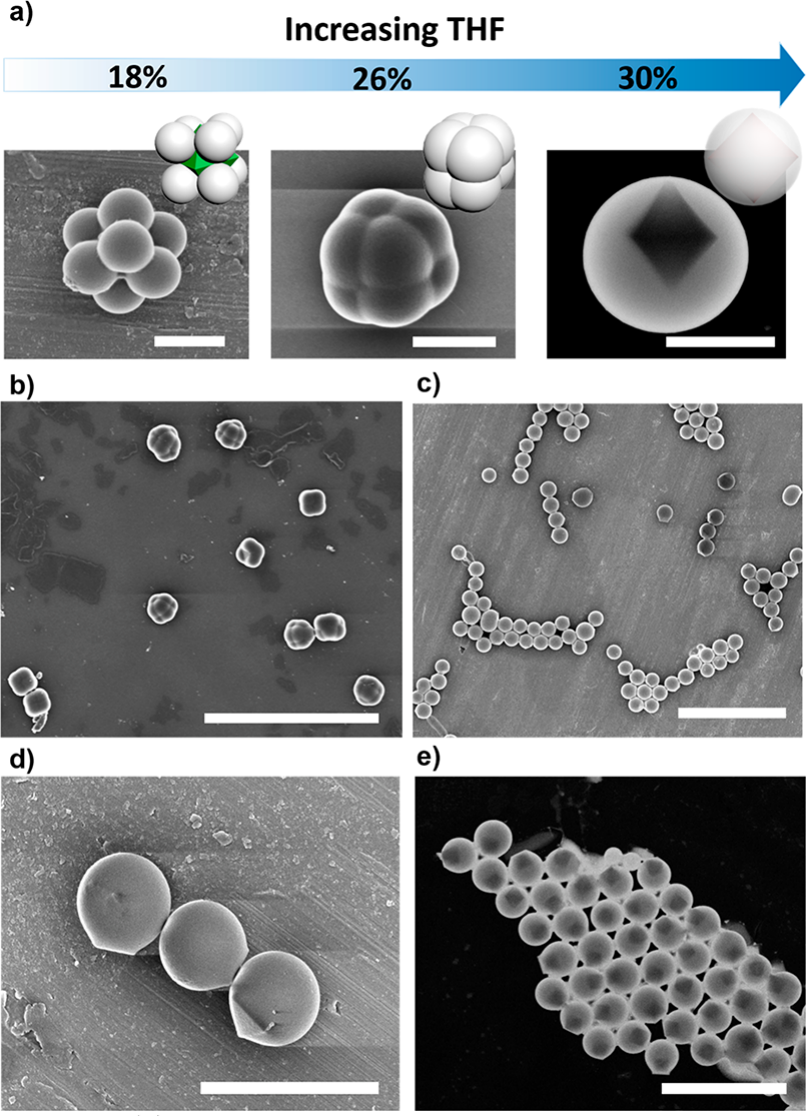
(a) Schematic illustration and corresponding FESEM and dark-field STEM images of the evolution from 8-c cubic colloidal clusters to core–shell particles upon increasing the concentration of THF. (b) FESEM image of the cellular-type particles synthesized at 26% v/v THF. (c–e) FESEM (parts c,d) and dark-field STEM (part e) images of spherical core–shell UiO-66@polystyrene particles synthesized at 30% v/v THF. Scale bars: 10 μm (parts b,c), 5 μm (part e), 3 μm (part d), and 1 μm (part a).

In conclusion, we have demonstrated that the attachment of polystyrene particles on each face of a polyhedral metal–organic framework (MOF) particle can be controlled at the single-particle level. This control opens the door to using the polyhedral shape of MOF particles to drive the formation of colloidal clusters. Within this strategy, both the coordination number and geometry of the colloidal cluster are defined by the type of polyhedron used as the core particle. As a proof of concept, we synthesized six-coordinated (6-c) octahedral, 8-c cubic, and 12-c cuboctahedral clusters using cubic ZIF-8, octahedral UiO-66, and rhombic dodecahedral ZIF-8 core particles, respectively. Moreover, we extended the use of this approach to create core–shell MOF@polymer particles in which a single MOF crystal that is used as the core particle is encapsulated in a polystyrene sphere. We believe that this assembly approach will open new avenues for the synthesis of novel colloidal clusters with unprecedented geometries, including those with high connectivity, as well as for increasing the repertoire of colloidal clusters to assemble new three-dimensional superlattices.

## Supporting Information Available

Detailed syntheses, FESEM data and images, as well as PXRD diagrams (PDF)
