# Supplementary material for: Assembly of Colloidal Clusters Driven by the Polyhedral Shape of Metal–Organic Framework Particles
Source: J Am Chem Soc. 2021 Aug 12;143(33):12943–7. doi: 10.1021/jacs.1c05363 (PMC8391935; doi:10.1021/jacs.1c05363)
Supplement: Supplementary file 1 — ja1c05363_si_001.pdf [file ja1c05363_si_001.pdf]

Supporting Information

Assembly of Colloidal Clusters Driven by the Polyhedral Shape of Metal-Organic Framework Particles

Yang Liu,[†] Jiemin Wang,[†] Inhar Imaz,[*†] and Daniel Maspoch[*†,‡]

[†]Catalan Institute of Nanoscience and Nanotechnology (ICN2), CSIC, and Barcelona Institute of Science and Technology, Campus UAB, Bellaterra, 08193 Barcelona, Spain
[‡]ICREA, Pg. Lluís Companys 23, Barcelona, 08010, Spain

Table of Contents

Materials S3
Synthesis of octahedral UiO-66 particles S3
Synthesis of cubic ZIF-8 particles S3
Synthesis of rhombic dodecahedral (RD) ZIF-8 particles S3
Synthesis of 8-coordinated cubic clusters S4
Synthesis of cellular-type particles and core-shell UiO-66@polystyrene particles S4
Synthesis of 6-coordinated octahedral clusters S4
Synthesis of 12-coordinated cuboctahedral clusters S5
Characterization S5
Figure S1 FESEM images and size-distribution histograms of octahedral UiO-66 particles S6
Figure S2 PXRD pattern of as-synthesized octahedral UiO-66 particles S7
Figure S3 FESEM images of 400 nm diameter of PS assembly with UiO-66 particles S8
Figure S4 FESEM images of 1 μm diameter of PS assembly with UiO-66 particles S9
Figure S5 FESEM images of the colloidal cubic clusters synthesized at the ratio of 40:1 .... S10
Figure S6 FESEM images of the colloidal cubic clusters synthesized at the ratio of 80:1 .... S11
Figure S7 FESEM images of the colloidal cubic clusters synthesized at the ratio of 150:1 .... S12
Figure S8 FESEM images of the colloidal cubic clusters synthesized at the ratio of 300:1 .... S13
Figure S9 DLS histogram of the size distribution S14
Figure S10 FESEM images and size-distribution histograms of cubic ZIF-8 particles S15
Figure S11 PXRD pattern of as-synthesized cubic ZIF-8 particles S16
Figure S12 FESEM images and size-distribution histograms of RD ZIF-8 particles S17
Figure S13 PXRD pattern of as-synthesized RD ZIF-8 particles S18
Figure S14 FESEM images of the 12-c cuboctahedral clusters etched by gradient solution S19
References S20

Experimental Section

Materials. Zirconium (IV) chloride ($ZrCl_4$), hexadecyltrimethylammonium bromide (CTAB), terephthalic acid (1,4-BDC) and polyvinylpyrrolidone (PVP, Mn=10,000) were purchased from Sigma Aldrich. Glacial acetic acid, tetrahydrofuran (THF) and *N*,*N*-dimethylformamide (DMF) were purchased from Fisher Chemical. 2-methylimidazole (2-MiM), zinc acetate dihydrate ($Zn(CH_3COO)_2{\cdot}2H_2O$), zinc nitrate hexahydrate ($Zn(NO_3)_2{\cdot}6H_2O$), sucrose and tetra-*n*-butylammonium bromide (TBAB) were purchased from TCI Chemical. Amino and sulfonated polystyrene (PS) particles were purchased from Thermo Fisher and Polysciences Europe. All chemical reagents and solvents were used as received without further purification. De-ionized (DI) water was obtained from a Milli-Q water purification system.

Synthesis of octahedral UiO-66 particles. $ZrCl_4$ (56 mg) and 1,4-BDC (40 mg) were dissolved in 20 mL of DMF containing 4 mL acetic acid and transferred to a scintillation vial. This mixture was heated at 120 °C for 12 h. Afterwards, the synthesized particles were washed twice with DMF (10 mL) and twice with methanol (10 mL) upon centrifugation at 9000 rpm in 50-mL Falcon tubes. Once washed, the collected particles were redispersed in 10 mL of an aqueous solution of PVP (2 mg mL^{-1}), stirred for 30 min, and washed once with 10 mL of DI water upon centrifugation at 9000 rpm in 50-mL Falcon tubes. The collected particles were finally redispersed in 5 mL of an aqueous solution of PVP (2 mg mL^{-1}) at a final concentration of UiO-66 particles of 10 mg mL^{-1}. The zeta potential of the resulting octahedral UiO-66 particles was approximately +45 mV. Note here that PVP was added to improve the colloidal stability of UiO-66 particles in water.

Synthesis of cubic ZIF-8 particles. In a typical synthesis, $Zn(NO_3)_2{\cdot}6H_2O$ (140 mg) dissolved in 8 mL of water was added to 24 mL of an aqueous solution containing 2-MiM (1920 mg) and 4 mL of 0.85 mg mL^{-1} CTAB solution. After standing for 5 hours, the resulting ZIF-8 particles were washed with deionized water (10 mL) upon centrifugation at 9000 rpm in 50-mL Falcon tubes, and finally dispersed in 10 mL of an aqueous solution of TBAB (1 mg/mL) at a final concentration of ZIF-8 particles of 10 mg mL^{-1}. The zeta potential of the resulting cubic ZIF-8 particles was approximately +40 mV. Note here that TBAB was added to improve the colloidal stability of ZIF-8 particles in water.

Synthesis of rhombic dodecahedral (RD) ZIF-8 particles. RD ZIF-8 particles were produced by following the protocol reported in a previous work.[1] An aqueous solution (5 mL) of $Zn(CH_3COO)_2{\cdot}2H_2O$ (300 mg) was added to 5 mL of an aqueous solution of 2-MiM (1.12 g) with gentle stirring. Then, the mixture was left at room temperature for 24 h. The resulting ZIF-8 particles were washed with deionized water (10 mL) upon centrifugation at 9000 rpm in 50-mL Falcon tubes, and finally dispersed in 10 mL of an aqueous solution of TBAB (1 mg/mL) at a final concentration of ZIF-8 particles of 20 mg mL^{-1}. The zeta potential of the resulting RD ZIF-8 particles was approximately + 40 mV. Note here that TBAB was added to improve the colloidal stability of ZIF-8 particles in water.

Synthesis of 8-coordinated cubic clusters using octahedral UiO-66 particles. In general, colloidal clusters were assembled by i) rapid addition of negatively charged polystyrene spheres suspension to an equal volume of suspension with monodispersed positively charged MOF particles; immediately addition of a water–soluble plasticizer (THF) to the resulting suspension; and iii) mixing and shaking the suspension by hand within 10 seconds. Specifically, for the 8-coordinated cubic clusters, they were assembled by adding 100 µL of the PS colloid (700 nm, 150 mg mL^{-1}) into 100 µL of the octahedral UiO-66 suspension (1 mg mL^{-1}). Then, 400 µL of THF/H_2O (18 % v/v) were rapidly added to the mixture, followed by hand shaking for 10 seconds. After that, the as-obtained sediments of the mixture suspensions were collected by further purification via density gradient centrifugation (10-30 wt% sucrose in water) and washed with deionized water for several times. The resulting colloidal clusters were finally re-dispersed in DI water.

Note here that the density gradient centrifugation is a common technique used to separate particles that have different density or size of components. In it, the mixture is placed into a centrifuge, in which the spinning from the centrifuge causes more dense particles to move to the outside edge, creating a sorted solution that is layered by particle density from least to most. In this technique, density gradient reagents (usually glycerol, sucrose and caesium chloride) are used to speed up the process and increase the purity and throughput. In our experiments, we found that a density gradient of 10-40 wt% sucrose in water was optimum to purify the mixture.

Synthesis of cellular-type particles and core-shell UiO-66@polystyrene particles using octahedral UiO-66 particles. The syntheses were identical to the formation of the above-mentioned 8-coordinated cubic clusters**,** except that these two types of particles were obtained by adding higher concentration of THF plasticizer: 400 µL of THF/H_2O (26% v/v)

for the synthesis of the cellular-type particles; and 400 μL of THF/H_2O solution (30% v/v) for the core-shell UiO-66@polystyrene particles.

Synthesis of 6-coordinated octahedral clusters using cubic ZIF-8. These colloidal clusters were assembled by adding 100 μL of the PS spheres colloid (200 nm, 10 mg mL^{-1}) into 100 μL of the cubic ZIF-8 particles colloid (3 mg mL^{-1}). Then, 350 μL of THF/H_2O (14 % v/v) were rapidly added to the mixture, followed by hand shaking for 10 seconds. After standing for 10 minutes without disturbing, the upper supernatant of the mixture suspension was removed carefully while the sediments were collected. The collected solid was redispersed in DI water (2 mL) and this sedimentation-redispersion process was repeated three more times. Finally, the collected colloidal clusters were redispersed in DI water.

Synthesis of 12-coordinated cuboctahedral clusters using RD ZIF-8 particles. These colloidal clusters were assembled by adding 100 μL of the PS colloid (600 nm, 20 mg mL^{-1}) in 100 μL of the RD ZIF-8 particles colloid (3 mg mL^{-1}). Then, 400 μL of THF/H_2O (17 % v/v) were rapidly added to the mixture, followed by hand shaking for 10 seconds. After standing for 10 minutes without disturbing, the upper supernatant of the mixture suspension was removed carefully while the sediments were collected. The collected solid was redispersed in DI water (2 mL) and this sedimentation-redispersion process was repeated three more times. Finally, the collected colloidal clusters were redispersed in DI water.

Characterization. Field-emission scanning electron microscopy (FE-SEM) images were collected on a scanning electron microscope (FEI Magellan 400L XHR) at acceleration voltage of 2.0 kV, using aluminum-tape or silicon wafer as support. Bright or dark field scanning transmission electron microscopy (STEM) images were obtained with scanning electron microscope (FEI Magellan 400L XHR) at acceleration voltage of 20.0 kV. The average size distributions of synthesized particles/clusters were statistically estimated from FE-SEM images by counting the edge/diagonal of 200 particles/clusters at different areas from one sample. The diameter of UiO-66 particles was calculated from the previously measured edge size. PXRD measurements were carried out on an X'Pert PRO MPDP analytical diffractometer, λ_{Cu} = 1.5406 Å (PANalytical). The surface charge of MOF particles (expressed as zeta potential (ζ)) was measured by using a Malvern Zetasizer, (Malvern Instruments, UK).

a)

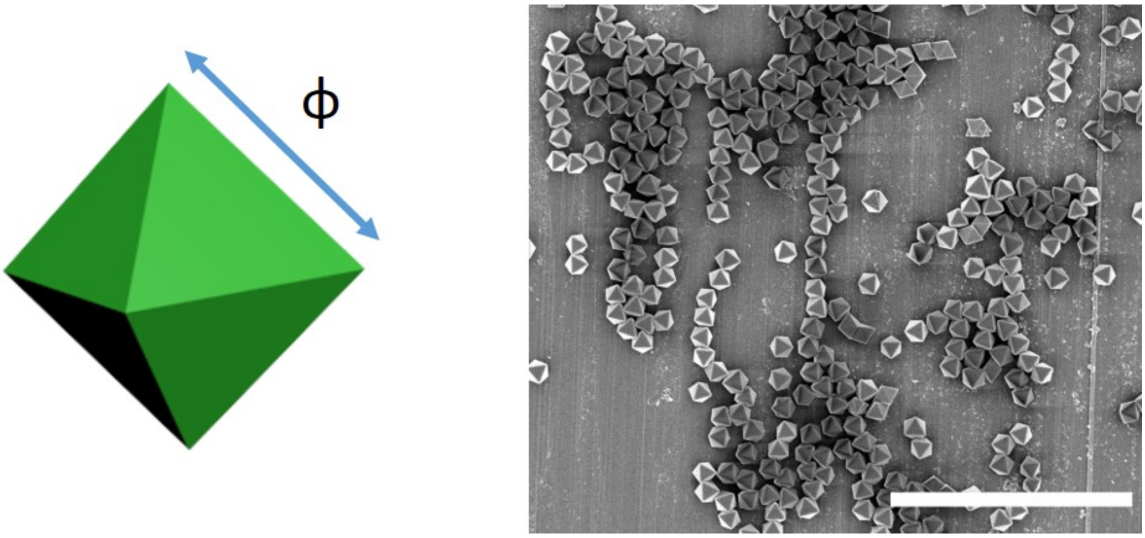

b)

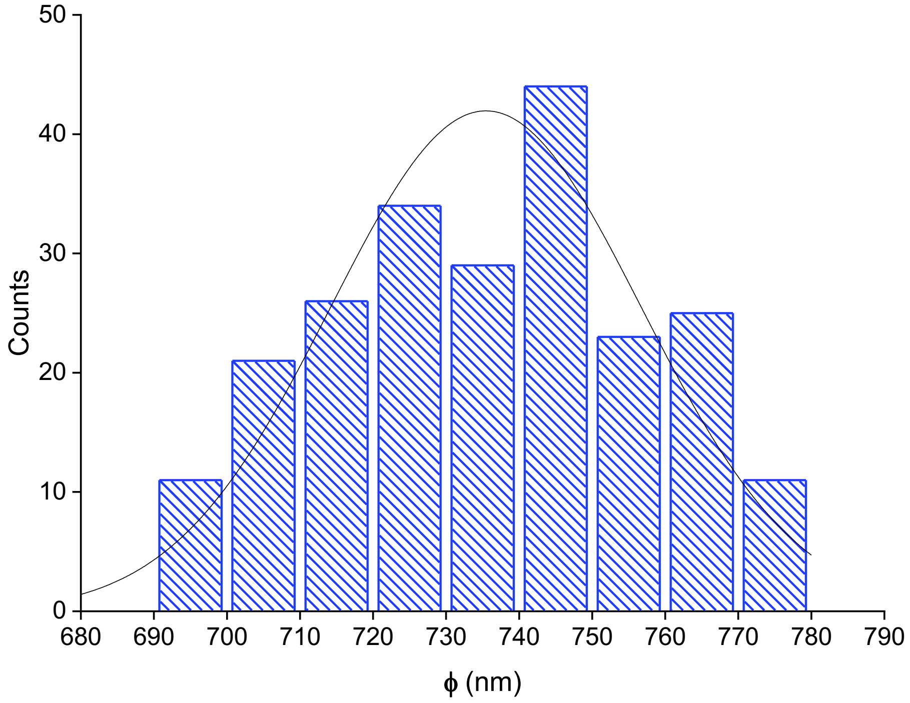

Figure S1. (a) Scheme and FESEM image of as-synthesized octahedron UiO-66 particles, highlighting the edge length of particles (ϕ). Scale bar: 10 μm. (b) Size-distribution histogram of as-synthesized UiO-66 particles with a mean ϕ of 735±21 nm.

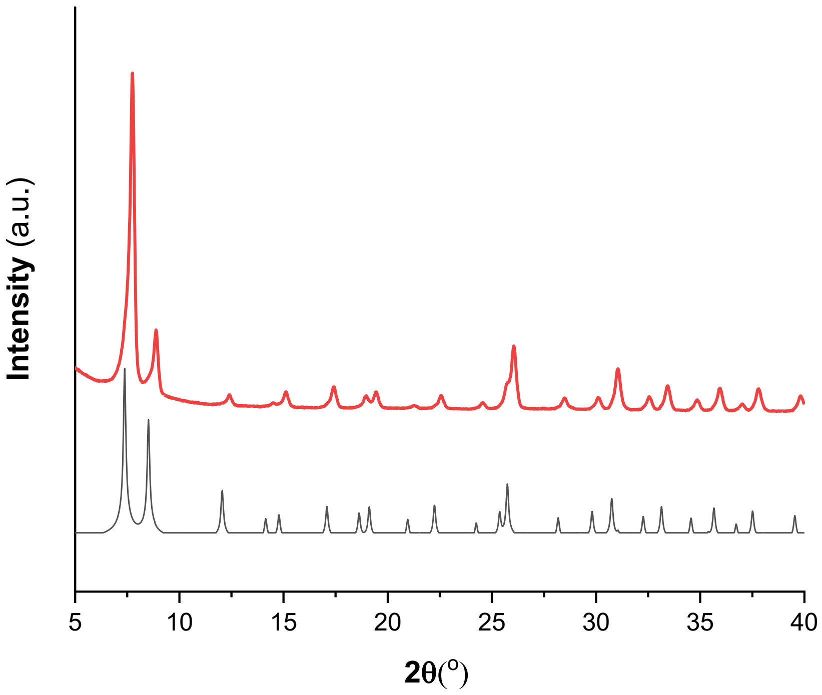

Figure S2. XRPD pattern of simulated (black) and as-synthesized UiO-66 particles (red).

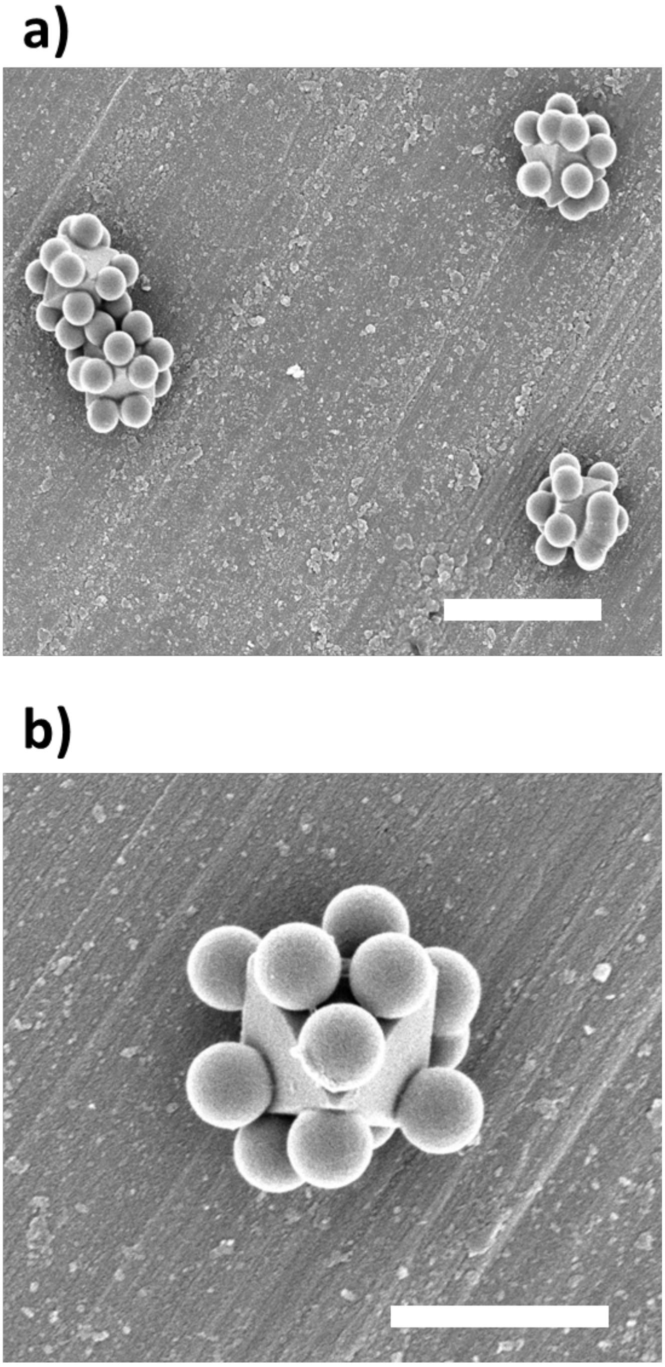

Figure S3. Representative FESEM images corresponding to the experiment in which 8-c cubic clusters were assembled by combining octahedron UiO-66 particles with 400 nm-in-diameter of polystyrene spheres. Scale bars: (a) 5 μm and (b) 1 μm.

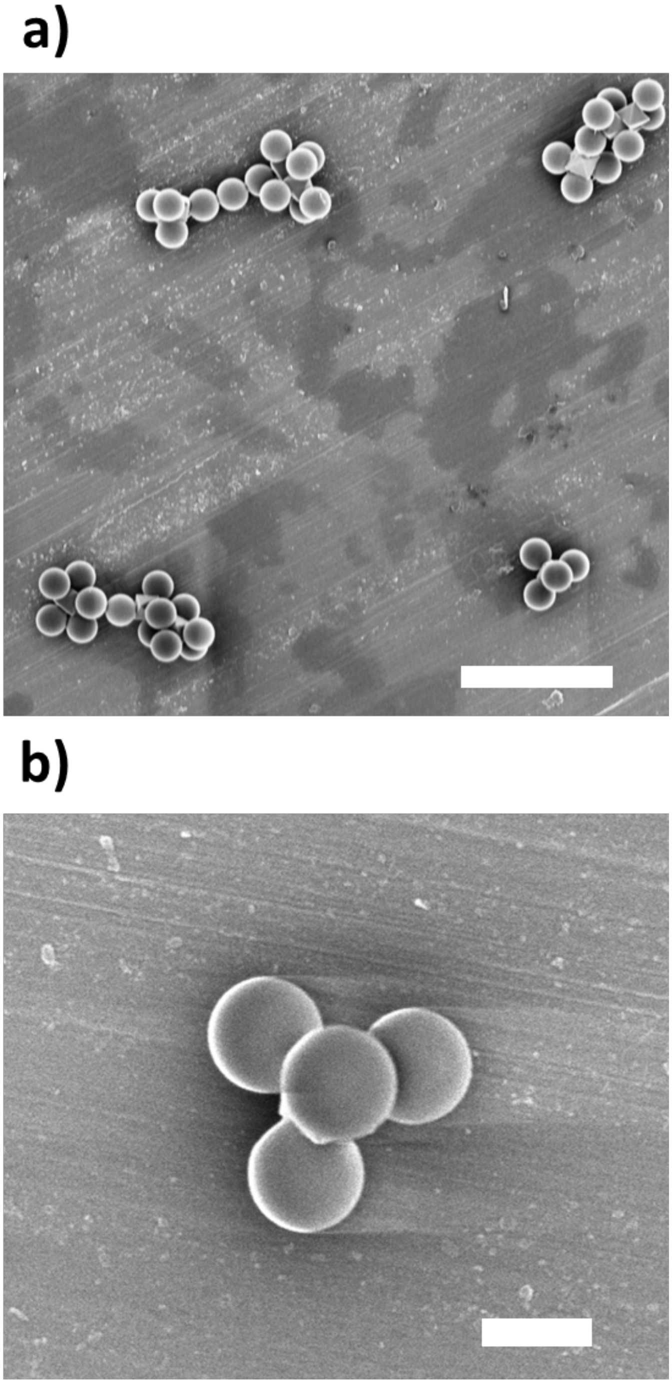

Figure S4. Representative FESEM images corresponding to the experiment in which 8-c cubic clusters were assembled by combining octahedron UiO-66 particles with 1 μm-in-diameter of polystyrene spheres. Scale bars: (a) 5 μm and (b) 1 μm.

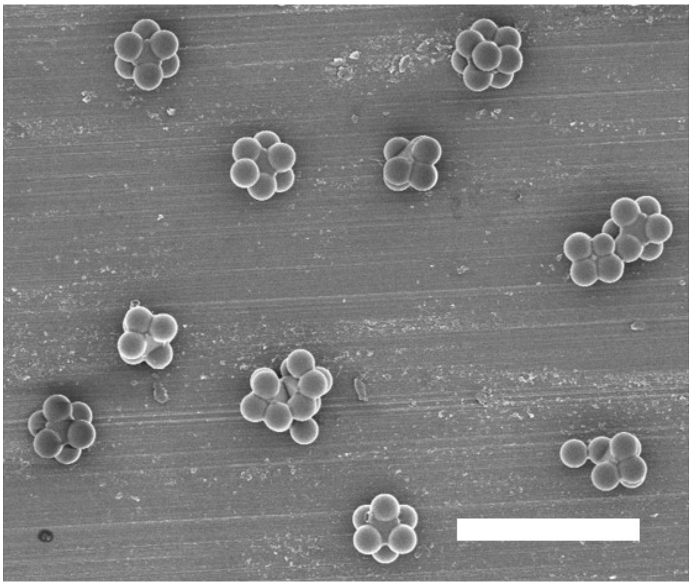

Figure S5. Representative FESEM image of the colloidal cubic clusters synthesized at the ratio of 40:1. Scale bar: 10 μm.

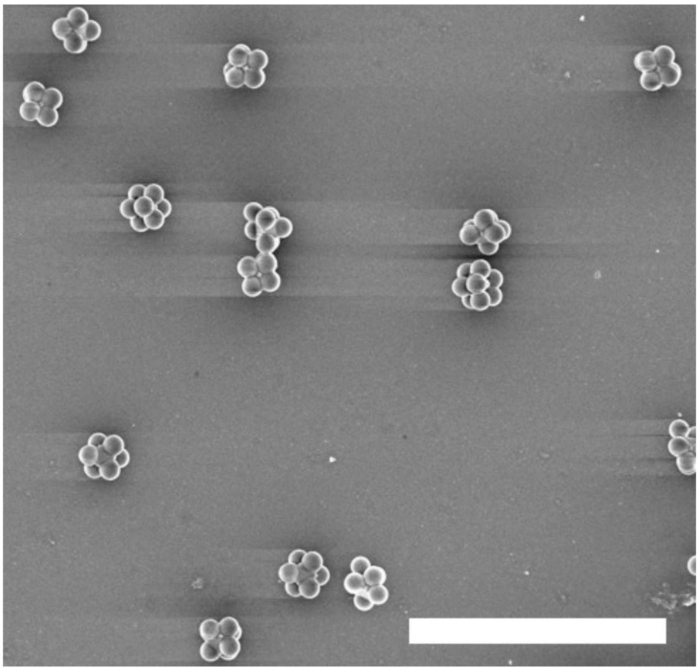

Figure S6. Representative FESEM image of the colloidal cubic clusters synthesized at the ratio of 80:1. Scale bar: 10 μm.

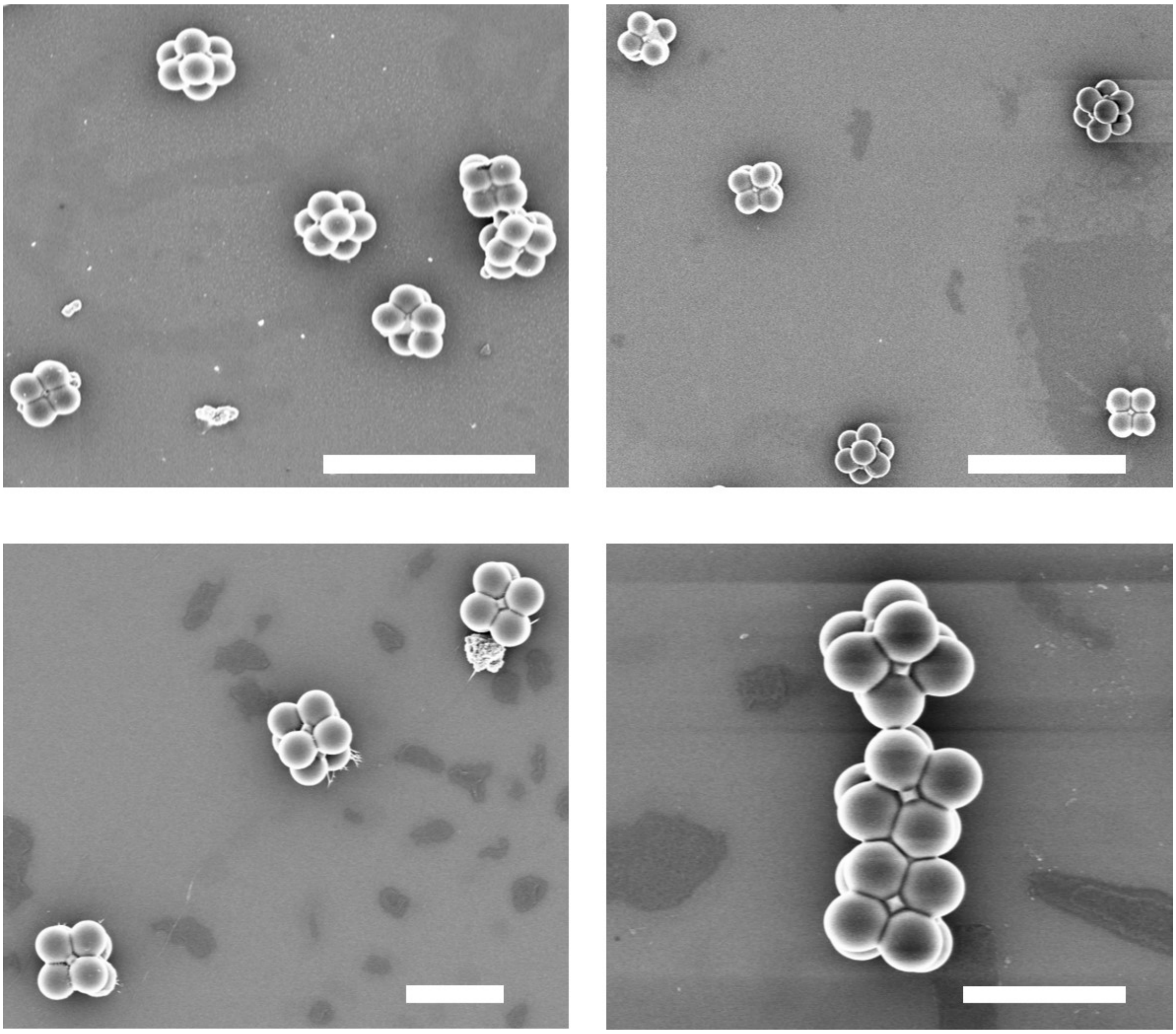

Figure S7. Representative FESEM images of the colloidal cubic clusters synthesized at the ratio of 150:1. Scale bars: (top row) 5 μm and (bottom row) 2 μm.

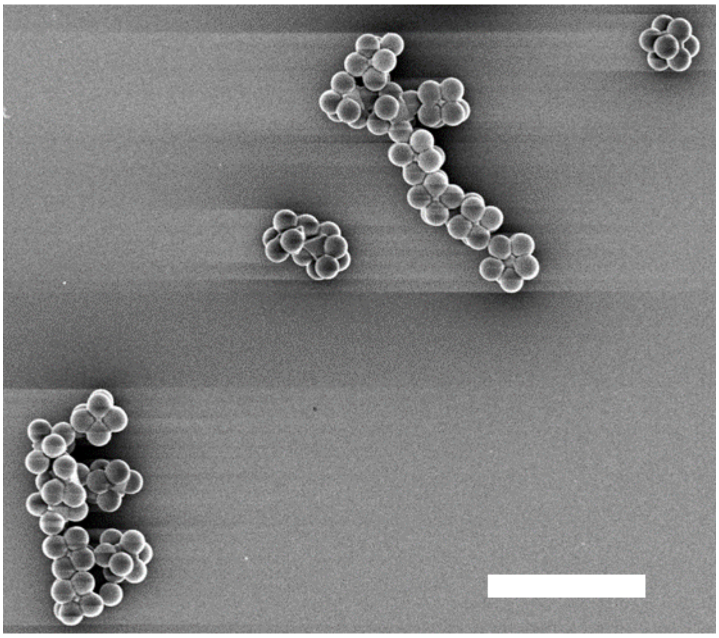

Figure S8. Representative FESEM image of the colloidal cubic clusters synthesized at the ratio of 300:1. Scale bar: 5 μm.

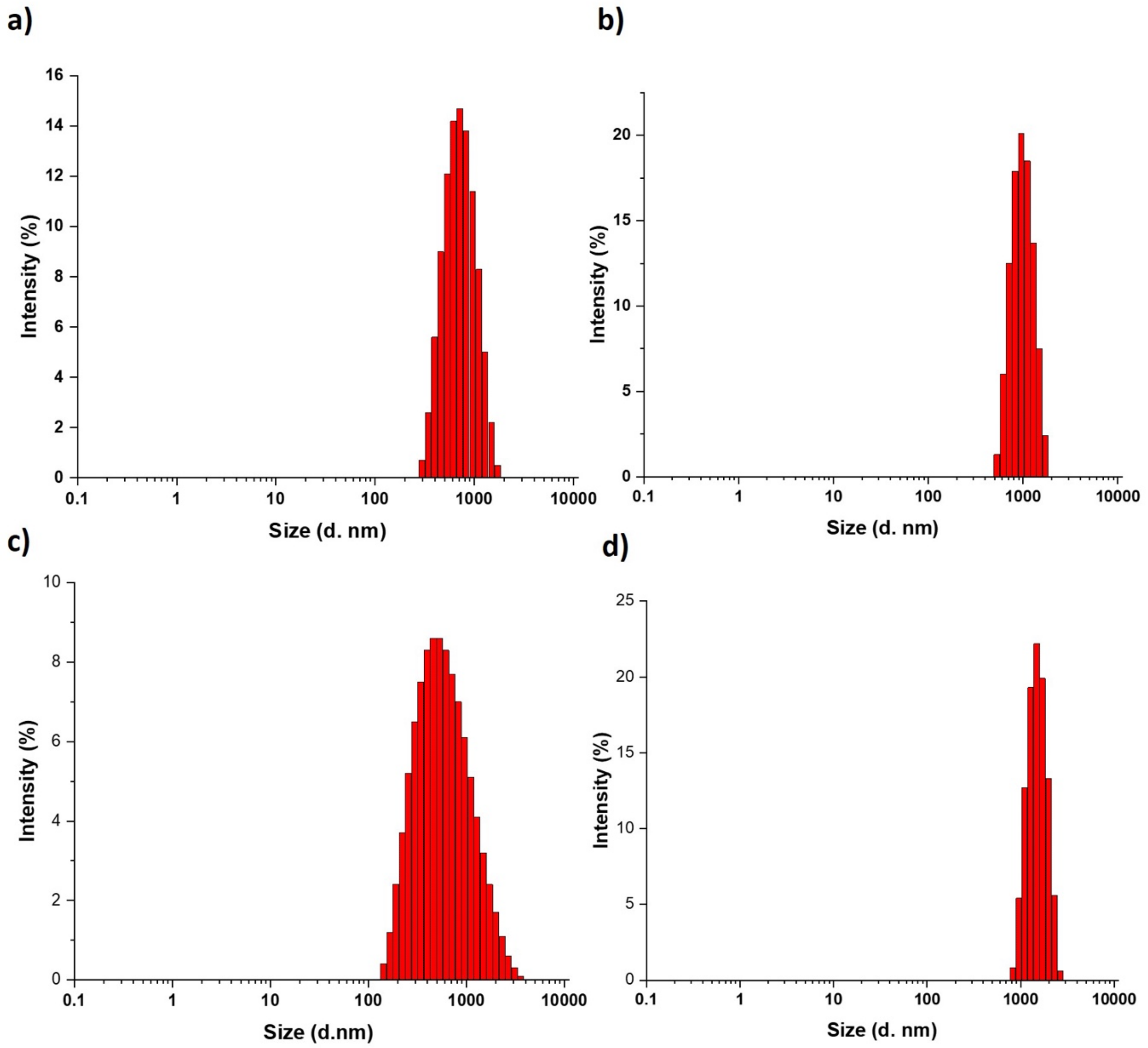

Figure S9. DLS histogram of the size distribution of a) polystyrene spheres (mean average size: 782 ± 31 (PDI: 0.163)； b) UiO-66 particles (mean average size: 1022 ± 20 (PDI: 0.098)； c) the mixture of polystyrene spheres and UiO-66 particles (mean average size: 717 ± 30 (PDI: 0.328)； and d) purified colloidal cubic clusters (mean average size: 1453 ± 73 nm (PDI: 0.385). Note that the later mean size measured by DLS matches with the size of the cubic clusters measured by FESEM.

a)

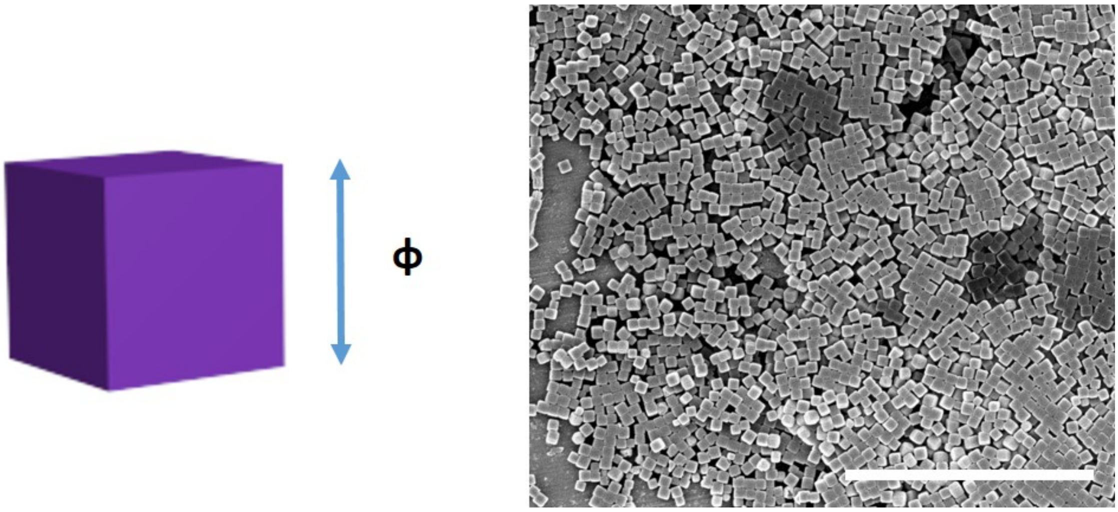

b)

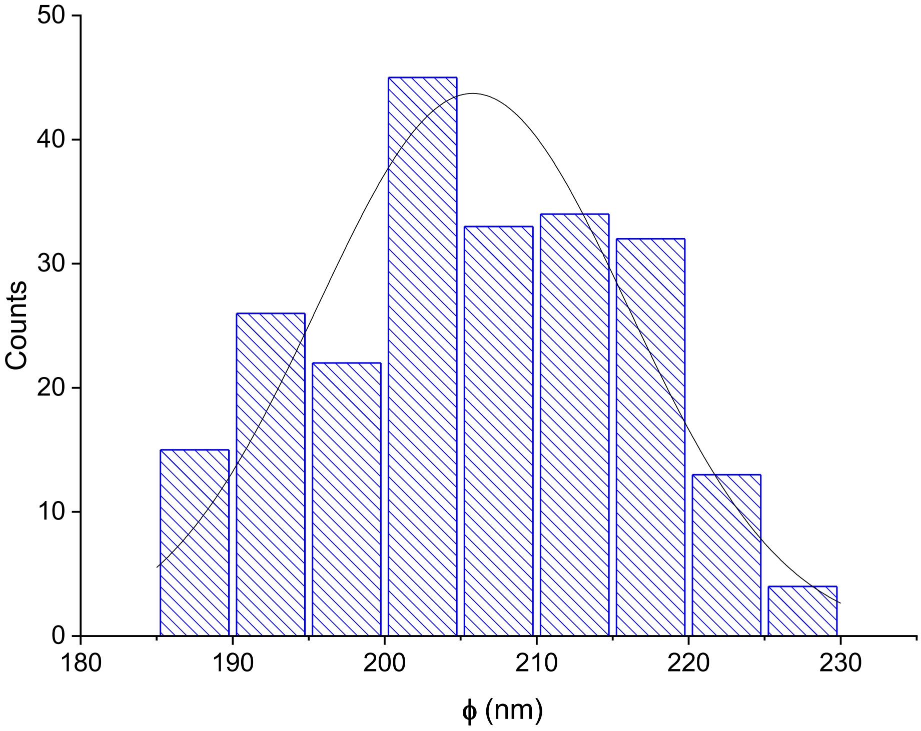

Figure S10. (a) Scheme and FESEM image of as-synthesized cubic ZIF-8 particles, highlighting the edge length of particles (ϕ). Scale bar: 5 μm . (b) Size-distribution histogram of as-synthesized cubic ZIF-8 particles with a mean ϕ of 205±10 nm.

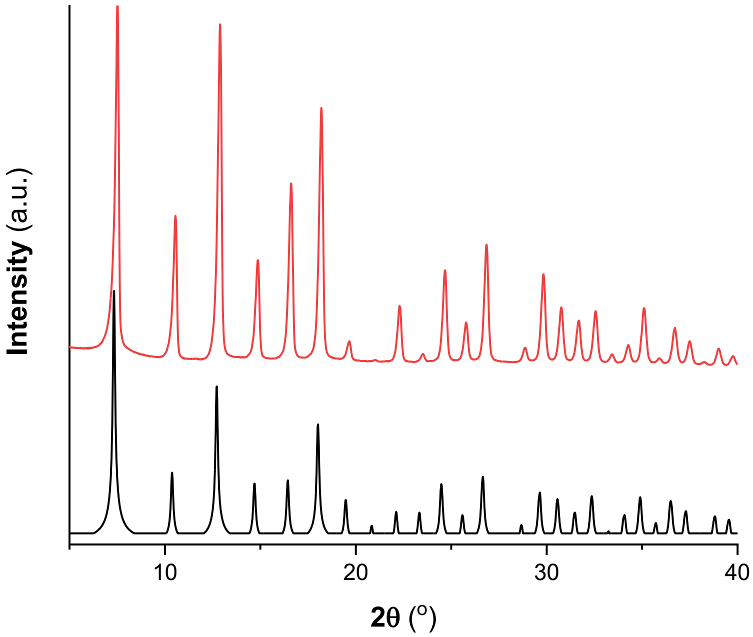

Figure S11. XRPD pattern of simulated (black) and as-synthesized cubic ZIF-8 particles (red).

a)

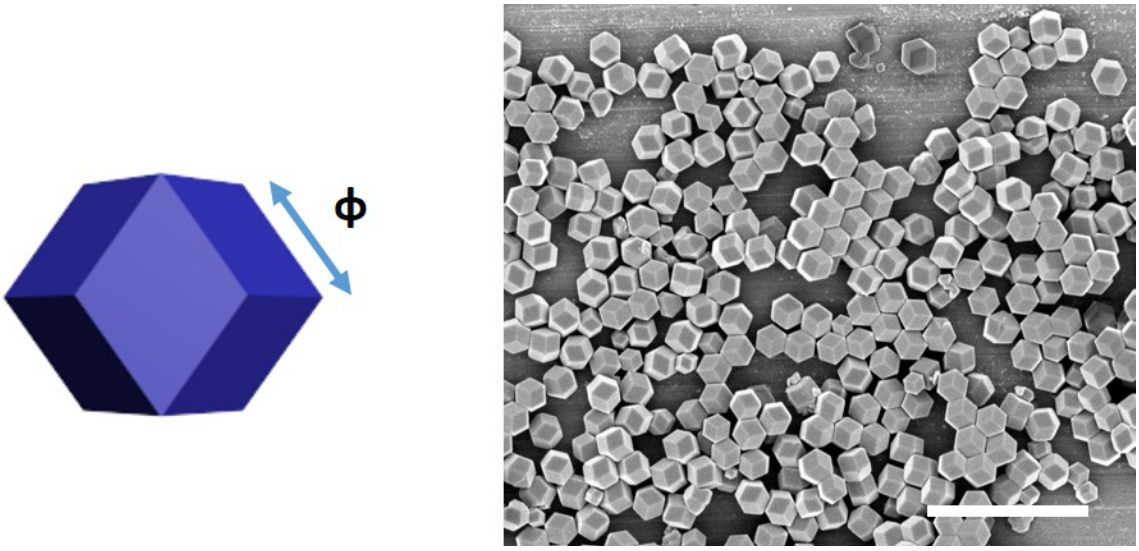

b)

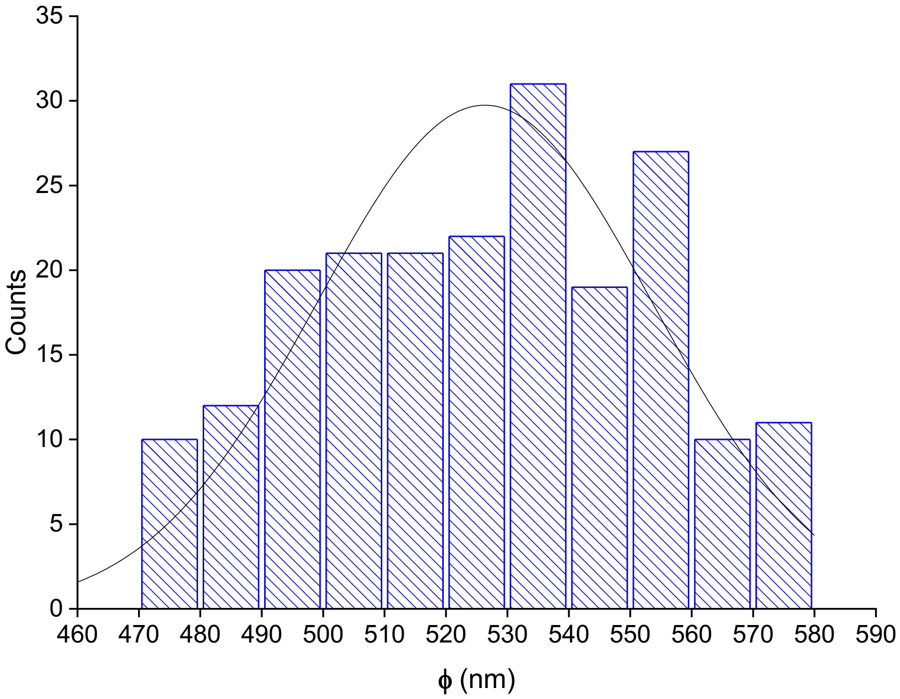

Figure S12. (a) Scheme and FESEM image of as-synthesized RD ZIF-8 particles, highlighting the edge length of particles (ϕ). Scale bar: 5 μm. (b) Size-distribution histogram of as-synthesized RD ZIF-8 particles with a mean ϕ of 526±27 nm.

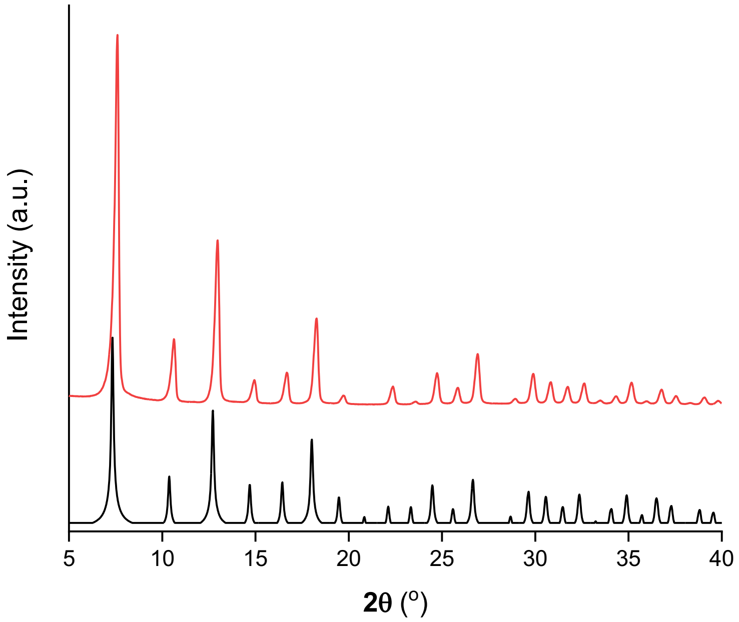

Figure S13. XRPD pattern of simulated (black) and as-synthesized RD ZIF-8 particles (red).

a)

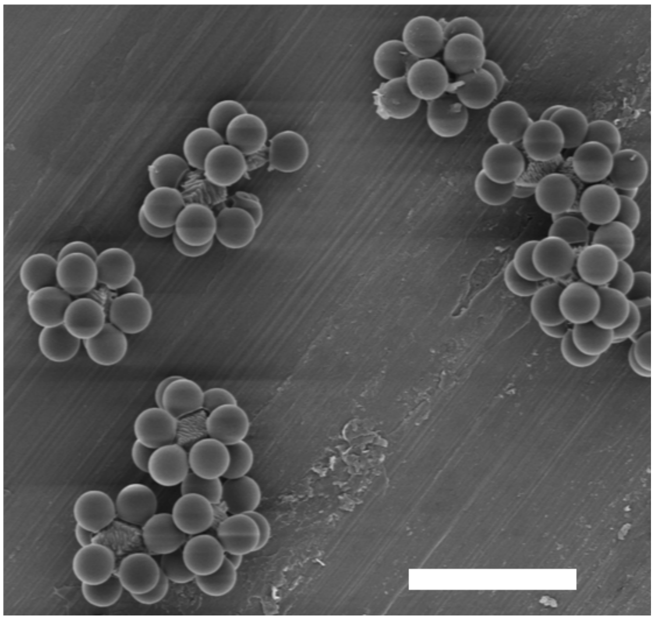

b)

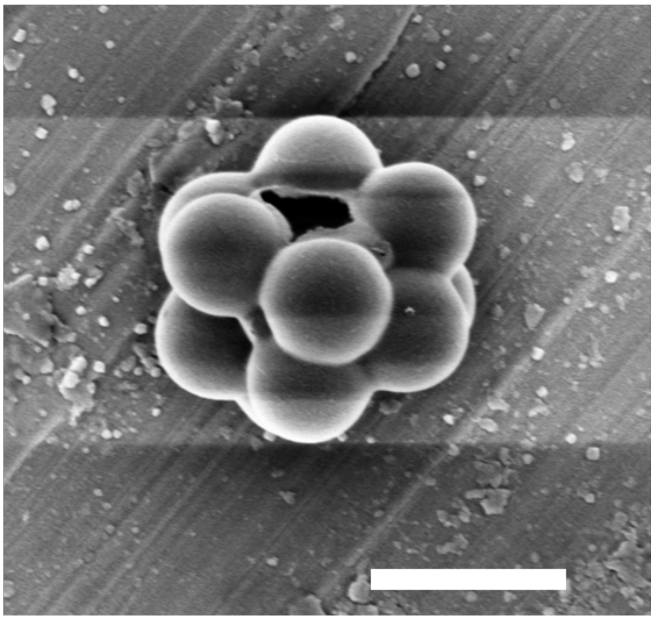

Figure S14. Representative FESEM images of 12-c cuboctahedron clusters, in which the central ZIF-8 particles have been etched by the gradient solution. Scale bars: (a) 5 μm and (b) 1 μm.

References

1. Avci, C.; Ariñez-Soriano, J.; Carné-Sánchez, A.; Guillerm, V.; Carbonell, C.; Imaz, I.; Maspoch, D. Post-Synthetic Anisotropic Wet-Chemical Etching of Colloidal Sodalite ZIF Crystals. *Angew. Chem., Int. Ed.* **2015**, 54(48), 14417-14421.
